# Female Functional Constipation Is Associated with Overactive Bladder Symptoms and Urinary Incontinence

**DOI:** 10.1155/2017/2138073

**Published:** 2017-02-28

**Authors:** Takahiro Maeda, Masuomi Tomita, Atsushi Nakazawa, Gen Sakai, Shinsuke Funakoshi, Akari Komatsuda, Yujiro Ito, Hirohiko Nagata, Nobuhiro Tsukada, So Nakamura

**Affiliations:** ^1^Department of Urology, Saiseikai Central Hospital, Tokyo, Japan; ^2^Department of Internal Medicine, Saiseikai Central Hospital, Tokyo, Japan

## Abstract

This noninterventional cross-sectional study aims to assess the association between functional constipation (FC) and urinary symptoms in female patients with no treatment for urination and defecation. The Rome III criteria for evaluation of defecation, Overactive Bladder Symptom Score (OABSS) for evaluation of urinary symptoms, and clinical features were investigated in 145 female patients. Latent FC and moderate to severe overactive bladder (OAB) were defined on the basis of positivity for two or more of the Rome III criteria and an OABSS ≥ 6 with OABSS Q3 ≥ 2, respectively. In 60 latent FC patients, the OABSS was higher (5.0 versus 3.2, *p* = 0.001), and concurrent moderate to severe OAB symptoms and OAB with urinary incontinence were more frequent than those in 85 nonlatent FC patients (33.3 versus 10.6%, *p* = 0.001, and 31.7 versus 7.1%, *p* < 0.001). Multivariate analysis demonstrated that moderate to severe OAB symptoms were a significant associated factor of latent FC (odds ratio (OR) = 4.125, *p* = 0.005), while latent FC was the only associated factor of moderate to severe OAB and OAB with urinary incontinence (OR = 4.227, *p* = 0.005 and OR = 4.753, *p* = 0.004). In conclusion, moderate to severe OAB symptoms are correlated with FC. Moreover, FC is related to moderate to severe OAB symptoms and to OAB with urinary incontinence.

## 1. Introduction

Overactive bladder (OAB) is defined as the symptom syndrome primarily composed of urinary urgency, usually with urinary frequency and nocturia, regardless of the existence of urgent incontinence [1]. According to a large-scale internet survey (EpiLUTS study), OAB affects mental health, work productivity [2, 3], and sexual health [4]. OAB is also associated with urinary tract infection, falls, and fractures [5]. Functional constipation (FC) also affects both physical and mental problems [6, 7], as well as being associated with the development of diverticular disease and colorectal cancer [8]. OAB and FC are common clinical problems in middle-aged and elderly women, both of which have an adverse influence on quality of life (QoL).

During fetal development, the bladder and intestines both arise from the embryologic hindgut. A close relationship between bladder function and intestinal function has been demonstrated in various animal models [9, 10] and in clinical studies [11, 12]. A urodynamic study by De Wachter et al. showed that rectal distention significantly influences the sensation of bladder filling [13], while Panayi et al. detected detrusor overactivity when the rectum was distended and not when it was empty [12]. Crosstalk between bladder and bowel might occur via overlapping neural pathways, including the dorsal root ganglia and spinal cord, and via shared neurotransmitters [14].

Some large cross-sectional surveys have indicated an association between bowel habits and OAB symptoms. Coyne et al. showed that women with OAB were significantly more likely to also have chronic constipation in a study of 2160 individuals aged ≥40 years from the United States [15], and Zang et al. also reported that constipation increased the risk of OAB based on their findings in 4684 individuals aged ≥20 years from China [16]. However, few studies have evaluated the association between constipation and the severity of OAB.

Therefore, we investigated the association between FC and various types of OAB, including moderate to severe OAB, wet OAB, and dry OAB.

## 2. Patients and Methods

### 2.1. Subject

During the study periods in 2014 to 2015, 817 female patients came to urological departments in our hospital. Of them, patients aged ≧40 years who had been on stable oral medication for at least 3 months were included in this study. Patients with a history of bladder cancer or colorectal cancer and patients on treatment on antimuscarinic drugs, beta 3 adrenoceptor agonists, or laxatives were not included because these diseases or drugs may influence urination or bowel activity. Written informed consents were obtained from 150 patients before inclusion, but we excluded 5 patients proved to take these urination medicines or laxatives from an interview sheet. Finally, this noninterventional cross-sectional study population consists of 145 women aged ≧ 40 years.

### 2.2. Study Design and Procedure

Primary objective of this study is to evaluate whether Overactive Bladder Symptoms Score (OABSS) of female patients with latent FC is higher than that of patients without latent FC at the urological department of the general hospital or not. For the secondary objectives are to investigate the associated factors for latent FC and mild to moderate OAB, wet OAB, and dry OAB. Sample size was determined by the free software of Vanderbilt University.

Urinary symptoms were evaluated by OABSS and constipation was evaluated by the Rome III criteria. The OABSS was developed to assess the presence and severity of OAB symptoms as a self-administered four-item questionnaire (score: 0 to 15) [17]. OAB was defined as OABSS ≥ 3 and Q3 ≥ 2 and it was classified into “wet OAB,” which was OAB with urinary incontinence, and “dry OAB,” which was OAB without urinary incontinence. OAB was also classified into three severity categories as follows: mild OAB (scores from 3 to 5), moderate OAB (6 to 11), and severe OAB (12 to 15). The Rome III criteria include six items related to defecation: straining, lumpy hard stools, sensation of incomplete evacuation, use of digital maneuvers, sensation of anorectal obstruction or blockage with 25 percent of bowel movements, and decrease in stool frequency (<three bowel movements per week). Latent FC was defined by positivity for two or more of the Rome III criteria [18]. The clinical background was also investigated, including the medical history (diabetes, hypertension, cardiovascular disease, and hyperlipidemia) and drug therapy (calcium antagonists, angiotensin-converting enzyme inhibitors, angiotensin II receptor blockers, antipsychotic drugs, and diuretics).

This study was conducted according to the Helsinki Declaration and the ethical guidelines established for clinical studies by the Ministry of Health, Labour and Welfare of Japan. It was approved by the ethics committee of Saiseikai Central Hospital.

### 2.3. Statistical Analysis

Results were presented as the mean ± standard deviation. We evaluated the association between latent FC and clinical background factors. Student's unpaired* t*-test was used for comparing continuous variables while the *χ*^2^ test was employed for comparisons between categorical variables. Logistic regression analysis was performed to calculate odds ratios (OR) for latent FC or each type of OAB. A* p* value less than 0.05 was accepted as significant. All analyses were performed using SPSS software (version 22; Stata Corp, Texas, USA).

## 3. Results

### 3.1. Comparison of Clinical Features between the Latent FC Group and Non-FC Group

The characteristics of the subjects are shown in Table 1. The age of the 145 patients was 68.6 ± 10.8 years. The latent FC group consisted of 60 patients (41.4%), and the non-FC group included 85 patients (58.6%). The OABSS of all patients was 3.9 ± 3.0. OABSS in patients aged 40–49, 50–59, 60–69, 70–79, and 80- were 2.9 ± 1.7, 1.9 ± 1.4, 4.2 ± 3.1, 3.6 ± 2.0, and, 5.6 ± 4.0, respectively. Among the145 patients, OAB was detected in 35 patients (24.1%), including 10 patients with dry OAB (OAB without urinary incontinence) and 25 patients with wet OAB (OAB with urinary incontinence). OAB was mild in 6/35 patients (17.1%), moderate in 24 patients (68.6%), and severe in 5 patients (14.3%). Twenty-one of the 145 patients (14.5%) had diabetes mellitus, 60 patients (41.4%) had hypertension, 18 patients (12.4%) had ischemic heart disease, and 33 patients (22.8%) had hyperlipidemia.

The percentage of OAB patients was higher in the latent FC group than in the non-FC group (36.7% versus 15.3%, *p* < 0.001). Concurrent moderate to severe OAB (OABSS ≥ 6 and Q3 ≧ 2) and wet OAB were more frequent in the latent FC group than in the non-FC group (*p* = 0.001 and *p* < 0.001, resp.). However, there was no significant difference of patients with dry OAB between the two groups (*p* = 0.449). There were no significant differences of past medical history, but use of antipsychotic drug was higher in the latent FC group (*p* = 0.015).

### 3.2. Associated Factors for Latent FC

The results of univariate and multivariate analyses performed to identify associated factors for latent FC are shown in Table 2. In univariate analysis, antipsychotic drugs and OABSS ≥ 6 were significantly associated with latent FC. Multivariate analysis demonstrated that antipsychotic drug use was a marginal association of latent FC (*p* = 0.071), while OABSS ≥ 6 was a significant association of latent FC (OR = 4.125, 95% CI 1.531–11.118, *p* = 0.005).

### 3.3. Independent Indicators of Moderate to Severe OAB, Wet OAB, and Dry OAB

Table 3 indicates the results of univariate and multivariate analysis for associated factors with moderate to severe OAB symptoms. Diabetes, use of calcium antagonists, use of antipsychotic drug, and latent FC were significant indicators for moderate to severe OAB symptoms in univariate analysis, while multivariate analysis indicated that latent FC was a significant relation of moderate to severe OAB symptoms (OR = 4.227, 95% CI 1.554–11.495, *p* = 0.005). We also divided OAB patients into wet and dry OAB to analyze the factors identifying each type. Multivariate analysis demonstrated that latent FC was the sole related factor of wet OAB (OR = 4.753, 95% CI 1.624–13.905, *p* = 0.004) (Table 4), but latent FC was not associated with dry OAB.

### 3.4. Comment

This study was conducted to investigate the association between latent FC and various types of OAB, including moderate to severe OAB, dry OAB, and wet OAB, in female patients aged ≥40 years attending the urology department in the real-world clinical setting. In the latent FC group, the OABSS was higher and there was an increased prevalence of moderate to severe OABSS and wet OAB compared with the non-FC group. Moderate to severe OAB symptoms were a significant association of latent FC. On the other hand, latent FC was independently associated with moderate to severe OAB symptoms or OAB accompanied by urinary incontinence. However, latent FC is not related to OAB without urinary incontinence.

These results might suggest a close association between the presence of latent FC and the urinary status, especially OAB symptoms. In fact, three large-scale cross-sectional studies on the association between constipation and OAB symptoms have been published [15, 16, 19]. Coyne et al. conducted an Internet survey of chronic constipation, fecal incontinence, and OAB symptoms in female patients aged ≥ 40 years. They reported that chronic constipation and fecal incontinence were more common in OAB patients and that defecation disorders were observed in about half of female OAB patients with incontinence [15]. In that study, OAB was diagnosed from only one question about the presence of urinary urgency and they did not assess the severity of OAB. In the report on Chinese women by Zhang et al., constipation was a significant predictor of dry OAB (OR = 3.92), but not wet OAB (OR = 1.02) [16]. In contrast, Moller et al. reported that constipation is a predictor of urinary urgency associated with incontinence (OR = 1.6) [19]. That finding was consistent with our results, but these two studies were conducted before the OABSS questionnaire was established in 2005 and the criteria for diagnosis of constipation were not described. The concept of constipation varies among patients and physicians [19]. Different questionnaires and criteria for OAB and FC might lead to different results. The questionnaires and definitions used in our study are well established and, to our knowledge, this is the first investigation of the correlation between FC and OAB severity in the real clinical setting using established questionnaires.

The pathophysiology underlying the association between FC and OAB symptoms is not well established, but Chen et al. reported that colorectal distention induced an increase of contractility like bladder distention and the vesicovascular reflex in an animal study [20]. Based on these results, one possible reason for the relation between bowel and bladder symptoms is that chronic distention of the colorectum induced by constipation or chronic high abdominal muscle pressure at defecation restricts spontaneous bladder distention and worsens irritative bladder symptoms.

Antimuscarinic drug therapy is commonly used for OAB symptoms, but these drugs often induce constipation and this influences the adherence rate [21]. Understanding the association between constipation and OAB symptoms could lead to optimized treatment for patients with either or both sets of symptoms. Based on our results that moderate to severe OAB is associated with latent constipation, we need to check not only urinary symptoms but also defecation habit when treating the female OAB patients. Moreover, we should consider prescription of OAB treatment medicines with a little effect on bowel function for these patients because recent antimuscarinic drugs cause a lower incidence of constipation [22], and there are also *β*3-adrenoreceptor agonists and transdermal patch drugs as effective as oral drugs that minimize adverse effects [23, 24].

There were several limitations of this study. First, the number of patients was not large and selection bias may have been introduced by investigating patients from the urology department, but the primary goal of this study was to focus on the association between latent FC and OAB symptoms in the real clinical setting. Also, other urinary factors (such as urodynamic studies, post-voiding residual urine, frequency-volume charts, and the existence of stress incontinence or overflow incontinence) or other QoL-related questionnaires were not evaluated. Thus, future studies should include these factors in order to better understand the association between FC and OAB symptoms.

## 4. Conclusion

OAB is more severe in patients with latent FC and FC is a significant predictor of moderate to severe OAB or wet OAB. It might be worthwhile to consider assessment of the defecation status in OAB patients.

## Figures and Tables

**Table 1:** Patient characteristics.

	Overall	Latent constipation	No constipation	*p* value
	*N* = 145	*N* = 60 (41.4%)	*N* = 85 (58.6%)	
Age	68.6 ± 10.8	70.5 ± 10.5	67.2 ± 10.8	0.063
OABSS	3.9 ± 3.0	5.0 ± 3.6	3.2 ± 2.2	0.001
Moderate to severe OAB	29 (20.0)	20 (33.3)	9 (10.6)	0.001
Dry OAB	10 (6.9)	3 (5.0)	7 (8.2)	0.449
Wet OAB	25 (17.2)	19 (31.7)	6 (7.1)	<0.001
Diabetes	21 (14.5)	11 (18.3)	10 (11.8)	0.268
Hypertension	60 (41.4)	28 (46.7)	32 (37.6)	0.277
Cardiovascular disease	18 (12.4)	7 (11.7)	11 (12.9)	0.819
Hyperlipidemia	33 (22.8)	12 (20.0)	21 (24.7)	0.745
Ca blocker	45 (31.0)	23 (38.3)	22 (25.9)	0.110
ACEI/ARB	29 (20.0)	10 (16.7)	19 (22.4)	0.399
Antipsychotic	7 (4.8)	6 (10.0)	1 (1.2)	0.015
Diuretic	9 (6.2)	3 (5.0)	6 (7.1)	0.613

Data are *n* (%) or mean SD.

OABSS: Overactive Bladder Symptom Score, OAB: overactive bladder, ACEI: angiotensin-converting enzyme inhibitor, and ARB: angiotensin II receptor blocker.

**Table 2:** Analysis of factors related to latent constipation.

Factor	Univariate analysis	Multivariate analysis	Odds ratio
	(*p* value)	(*p* value)	
Age < 70 versus ≥70	0.940	0.817	
Diabetes	0.268	0.891	
Hyperlipidemia	0.745	0.517	
Ca blocker	0.110	0.183	
ACEI/ARB	0.399	0.512	
Antipsychotic	0.015	0.071	
Diuretic	0.613	0.175	
Moderate to severe OAB	0.001	0.005	4.125 (1.531–11.118)

ACEI: angiotensin-converting enzyme inhibitor, ARB: angiotensin II receptor blocker, and OABSS: Overactive Bladder Symptom Score.

**Table 3:** Analysis of factors related to moderate to severe OAB.

Factor	Univariate analysis	Multivariate analysis	Odds ratio
	(*p* value)	(*p* value)	
Age < 70 versus ≥70	0.430	0.905	
Diabetes	0.025	0.228	
Hyperlipidemia	0.939	0.960	
Ca blocker	0.025	0.170	
ACEI/ARB	0.917	0.422	
Antipsychotic	0.012	0.441	
Diuretic	0.058	0.156	
Constipation	0.001	0.005	4.227 (1.554–11.495)

ACEI: angiotensin-converting enzyme inhibitor and ARB: angiotensin II receptor blocker.

**Table 4:** Analysis of factors related to wet OAB.

Factor	Univariate analysis	Multivariate analysis	Odds ratio
	(*p* value)	(*p* value)	
Age < 70 versus ≥70	0.350	0.651	
Diabetes	0.035	0.460	
Hyperlipidemia	0.912	0.673	
Ca blocker	0.013	0.098	
ACEI/ARB	0.998	0.473	
Antipsychotic	0.004	0.217	
Diuretic	0.187	0.544	
Constipation	0.001	0.004	4.753 (1.624–13.905)

ACEI: angiotensin-converting enzyme inhibitor and ARB: angiotensin II receptor blocker.

## Abbreviations

OAB: Overactive bladder
FC: Functional constipation
QoL: Quality of life
OABSS: Overactive Bladder Symptom Score
OR: Odds ratio.
